# From literature to biodiversity data: mining arthropod organismal traits with machine learning

**DOI:** 10.3897/BDJ.13.e153070

**Published:** 2025-08-05

**Authors:** Joseph Cornelius, Harald Detering, Oscar Lithgow-Serrano, Donat Agosti, Fabio Rinaldi, Robert M Waterhouse

**Affiliations:** 1 Dalle Molle Institute for Artificial Intelligence Research (IDSIA USI-SUPSI), Lugano, Switzerland Dalle Molle Institute for Artificial Intelligence Research (IDSIA USI-SUPSI) Lugano Switzerland; 2 SIB Swiss Institute of Bioinformatics, Lausanne, Switzerland SIB Swiss Institute of Bioinformatics Lausanne Switzerland; 3 Department of Ecology and Evolution, University of Lausanne, Lausanne, Switzerland Department of Ecology and Evolution, University of Lausanne Lausanne Switzerland; 4 Plazi, Bern, Switzerland Plazi Bern Switzerland; 5 Digital Society Initiative, University of Zurich, Zurich, Switzerland Digital Society Initiative, University of Zurich Zurich Switzerland

**Keywords:** arthropods, biodiversity, natural language processing, text and data mining, trait database

## Abstract

The fields of taxonomy and biodiversity research have witnessed an exponential growth in published literature. This vast corpus of articles holds information on the diverse biological traits of organisms and their ecologies. However, access to and extraction of relevant data from this extensive resource remain challenging. Advances in text and data mining (TDM) and Natural Language Processing (NLP) techniques offer new opportunities for liberating such information from literature. Testing and using such approaches to annotate articles in machine-actionable formats is, therefore, necessary to enable the exploitation of existing knowledge in new biology, ecology and evolution research. Here, we explore the potential of these methods to annotate and extract organismal trait data for the most diverse animal group on Earth, the arthropods. The article processing workflow uses manually curated trait dictionaries with trained NLP models to perform labelling of entities and relationships of thousands of articles. A subset of manually annotated documents facilitated the formal evaluation of the performance of the workflow in terms of entity recognition and normalisation and relationship extraction, highlighting several important technical challenges. The results are made available to the scientific community through an interactive web tool and queryable resource, the ArTraDB Arthropod Trait Database. These methodological explorations provide a framework that could be extended beyond the arthropods, where TDM and NLP approaches applied to the taxonomy and biodiversity literature will greatly facilitate data synthesis studies and literature reviews, the identification of knowledge gaps and biases, as well as the data-informed investigation of ecological and evolutionary trends and patterns.

## Introduction

The existing detailed knowledge on biodiversity and the natural world is contained largely in the form of an extensive and growing corpus of scientific publications (McCallen et al. 2019). This knowledge harbours important insights to better understand the dynamics and dimensions of major challenges facing our planet today, such as the global biodiversity crisis and the impact of climate change on the distribution of species. Large portions of that information have been difficult to access because they are unstructured, in printed formats, including portable data format (PDF), which have been difficult to machine operate or which are behind paywalled -ess. With increasing digitisation of the scientific publishing process, and thanks to comprehensive digitisation efforts of natural history collections (Hedrick et al. 2020), an increasing number of documents have become digitally accessible. For example, PubMedCentral (PMC) alone contains millions of machine actionable articles (Rosonovski et al. 2023), including millions of supplementary data files and tens of millions of abstracts are accessible through PubMed. These documents can be used for text and data mining (TDM) and natural language processing (NLP) to better annotate the literature, offering opportunities to liberate data and knowledge from publications. However, even though such literature mining approaches are recognised as important tools in biology, ecology and evolution research, their potential is currently far from fully realised (Farrell et al. 2022, Farrell et al. 2024).

To focus the methodological explorations of these opportunities, aiming to annotate and extract organismal trait data — such as habitat, ecology and morphology — for the most numerous and diverse animal group on Earth with estimates of 6.8 million terrestrial species (Stork 2018), the phylum Arthropoda represents an excellent case study. Arthropods have fascinated researchers and amateur entomologists for centuries, leading to a vast accumulation of knowledge about how countless evolutionary adaptations have enabled them to exploit so many ecological niches (Grimaldi and Engel 2005). Past research has often been biased towards those that serve as models in research, for example, the fruit fly or that impact human health and agriculture, for example, the malaria mosquito or the honeybee. The associated literature also present substantial variability in terms of what types of trait data have been collected and which methodologies were used (Wong et al. 2019). More recently, this biological knowledge is being extended through the acquisition of increasing amounts of genomics data to explore the genetics underlying these traits (Feron and Waterhouse 2022b). The motivation is to understand how genetic and genomic changes relate to observable phenotypic differences, i.e. traits, amongst species. Thanks to ongoing developments in bioinformatics, comparative genomics analyses are generally scalable to increasingly larger datasets, taking advantage of rapidly accumulating numbers of species with sequenced genomes (Feron and Waterhouse 2022a). However, this is not being matched by equivalent advances in the cataloguing of species traits for which manual collection and curation efforts cannot keep pace with the needs to access trait data for large-scale quantitative analyses. Literature mining offers potential solutions for overcoming these challenges, for example, building a database of insect egg size and shape for more than 6,700 species relied on information extracted from 1,756 publications (Church et al. 2019), cataloguing traits of 12,448 butterfly and moth species involved extracting information from 117 field guides and species accounts (Shirey et al. 2022) and compiling an expert-curated trait database of 520 subterranean spiders examined 255 taxonomic descriptions from the World Spider Catalog and the Spiders of Europe repository (Mammola et al. 2022). Therefore, efforts to develop systematic approaches for mining the literature to build comprehensive open databases of species’ organismal traits should finally enable researchers to access these large-scale biodiversity data.

Here, we present a TDM and NLP framework for the automated labelling of identified arthropod species (taxa), their organismal traits and the associated trait values in taxonomic and biodiversity research articles. We focus on PMC articles containing taxonomic treatments of arthropods, i.e. structured sections of publications that describe and define the name and features of species, leveraging the large resource of Plazi’s TreatmentBank (Guidoti et al. 2021). Using manually curated trait dictionaries, as well as subsets of manually annotated articles, we trained NLP models to perform labelling of entities (taxa, traits, values) and relationships (taxon to trait, trait to value) of thousands of articles. We formally evaluated the performance of our approaches, demonstrating their application to 2,000 publications, which produced 656,403 entity and 339,463 relationship annotations and highlighting several important technical challenges. Finally, we developed an interactive web tool that makes the results available to the scientific community in the form of the queryable database resource, the ArTraDB Arthropod Trait Database. Together, these tools and resources serve to advance the use of literature mining approaches in biology, ecology and evolution research, by semi-automating the building of comprehensive open databases of organismal traits extracted from literature.

## Materials and Methods

### Sourcing and Processing the Text Corpora

#### Articles Sourced from PubMedCentral via TreatmentBank

To take advantage of the normally highly structured and detailed species information found in taxonomic treatment texts and, at the same time, to reduce the complexity of the overall labelling process, the initial corpus of texts was defined using all arthropod species taxonomic treatment texts available from Plazi’s TreatmentBank (Guidoti et al. 2021). Taxonomic treatments refer to sections in scientific publications where the key features describing, distinguishing and naming a species are documented (Agosti et al. 2022). Treatments have been the building blocks of how data about taxa are provided ever since the beginning of modern taxonomy and usually follow a highly structured format. This simplifies the first task of labelling taxa (species) because each text already pre-processed by Plazi is directly linked to a known species, meaning at least one annotated taxon in the document should match the linked species name provided by TreatmentBank. From the ~ 310,000 treatment texts sourced from Plazi’s TreatmentBank, ~ 250,000 were linked to digital object identifiers (DOIs) comprising ~ 24,000 unique publications, 3,650 of which were linked with PubMedCentral (PMC) identifiers and thereby presented publicly accessible texts that could be used for labelling and subsequent mining. Note that publications may and often do, contain many treatments, i.e. descriptions of many species, hence the tenfold higher number of treatments compared to the number of publications.

#### Processing of PubMedCentral Articles

The PMC article files were retrieved in Extensible Markup Language (XML) format and subsequently transformed into plain text format, maintaining the original text extraction without further modifications such as lowercasing. For the Named Entity Recognition (NER) task, these PMC text files were subsequently converted into the CoNLL format (Kim Sang and De Meulder 2003) (a text file with one word per line with sentences separated by an empty line) using the IOB2 tagging scheme (Inside–Outside–Beginning) (Ramshaw and Marcus 1999). For the Relationship Extraction (RE) task, the same PMC text files were processed into a specialised JSON (JavaScript Object Notation) file format compatible with the “Language Understanding with Knowledge-based Embeddings” model (LUKE) (Yamada et al. 2020). This format splits the text up in context windows, which, by default, encompass six sentences, along with the offsets and labels for both the head and tail entities.

### Sourcing and Processing Taxonomy and Trait Data

#### Taxonomy Data Sourced from the Catalogue of Life

The Catalogue of Life (COL) represents an authoritative source of taxonomic data built and maintained through a long-term international collaboration of taxonomists and informaticians (Bánki et al. 2024). The COL was, therefore, selected as the reference taxonomy dataset for building a dictionary of arthropod taxa and for filtering the treatments to select only arthropod species for downstream processing. The monthly COL release of July 2022 (COL Version: 23-06-2022) was processed to extract all accepted taxa (dwc:taxonomicStatus == ‘accepted’) that are hierarchically below Arthropoda (dwc:taxonID == ‘RT’). The dictionary of arthropod taxa, therefore, contains all accepted arthropod species names along with the taxonomic lineage names ascending the species-genus-family-order-class hierarchy up to the phylum level of Arthropoda. This resulted in a dictionary containing a total of 1,015,642 species and 118,008 higher-level taxonomic names, for use as the input for downstream NER steps to label taxa identified in the processed documents. The COL processing scripts are available as part of the ATResourceManager Snakemake workflow (https://github.com/IDSIA-NLP/ATResourceManager).

#### Organismal Trait Data

No single, comprehensive, standardised and machine operable ontology of organismal traits was available to use to build a dictionary of arthropod trait data. Therefore, extensive manual curation of traits defined across several different resources was required to be as comprehensive as possible while leveraging existing resources and standards. Trait libraries were developed for three broad categories covering arthropod feeding ecology, habitat and morphology, always requiring that the trait was defined and/or described in an existing online resource with Uniform Resource Identifiers (URIs). The resources queried included: the Encyclopedia of Life (EOL) (Parr et al. 2014); the Environment Ontology (ENVO) (Buttigieg et al. 2016); the Relation Ontology (RO) (Mungall et al. 2023); the UBERON Anatomy Ontology (Mungall et al. 2012); the BRENDA Tissue Ontology (BTO) (Chang et al. 2021); as well as Wikidata, Wikipedia and Wiktionary for additional relevant terms with URIs that were not incorporated into a formal ontology. Trait names and definitions were inherited from the source ontologies/URIs. Traits were classified into types: “yes/no”, a taxon exhibits or does not exhibit the trait; “association”, one taxon is associated with another taxon through the trait; “measurement” for mass-related traits; “length/width” for measurable body parts; “count” for countable body parts. Synonyms of trait names were automatically generated by scraping synonyms from Synonyms.com and enhanced with word vectors from PubMed and the Common Core to obtain related terms. This approach was refined by implementing an improved search for synonyms, creating a new table that included only those synonyms appearing at least ten times in the 5,000 articles from the ZooKeys journal that were available in PMC. This method was devised to capture real words commonly used in taxonomic treatments, managing to generate synonyms for most terms. However, the synonyms contained instances of inaccurate or inappropriate terms so manual curation was applied to reduce redundancy and improve informativeness of the alternative phrasings including pluralisations. This resulted in a dictionary containing a total of 390 traits (81 feeding ecology; 184 habitat; 125 morphology, Suppl. material 1), to be used as the input for downstream NER steps to label traits identified in the processed documents.

### Curating Gold-Standard Annotation Data

To fine-tune and formally evaluate the performance of the NLP models employed for the NER and RE tasks (see below), two entomology domain experts annotated a set of 25 articles randomly selected from the 3,650 obtained from PMC. The two annotators employed the tagtog text annotation tool (Cejuela et al. 2014) that provides a user-friendly interface to manually annotate and normalise entities in documents imported from PMC, as well as to add entity labels, relationships and more. The annotators worked independently on their assigned documents to avoid biasing each other; however, they did develop a set of guidelines during the annotation process to describe the steps to follow for dealing with some more complex cases (Suppl. material 2). For example, in addition to the required entities “taxon”, “trait” and “value” and the required relationships “has_trait” and “has_value”, the entity “qualifier” and relationship “has_qualifier” were created in order to label and link taxonomic qualifiers to labelled taxa entities, such as “female”, “male”, “juvenile” or “larva”. To be able to compare annotator styles, a subset of five documents was independently annotated by both experts. The completed annotations of the 25 documents were exported from tagtog. Each document, originally in tagtog’s native HTML format accompanied by annotations in JSON format, was converted into a BioC JSON file (Comeau et al. 2013). This format serves as the base for all subsequent processes. The documents annotated by both experts were used to calculate the inter-annotator agreement by means of Cohen’s Kappa score (Cohen 1960). The agreement was evaluated by comparing exact and partial matches of named entities, as well as exact matches of relationships within a tolerance of four characters for the entity offset boundary in these documents. To be able to formally evaluate the performance of the NLP models, the gold-standard annotation data were randomly split into a training (TRAIN-GOLD) and an evaluation (TEST-GOLD) dataset (Suppl. material 3). The TRAIN-GOLD dataset contained four documents (812 entities, 641 relationships) and the TEST-GOLD dataset contained 21 documents (4,272 entities, 3,439 relationships), corresponding to a 1,453/7,711 (18.8%) ratio between TRAIN-GOLD and TEST-GOLD. The data split was deliberately chosen, opting for a larger TEST-GOLD dataset to ensure it is statistically robust for valid analysis. Simultaneously, the TRAIN-GOLD dataset was intentionally kept small enough to train NER and RE models under a low-resource setup.

### Natural Language Processing for Entity and Relationship Annotation

For automatic identification of arthropod trait value triples, a two-step pipeline was developed: first, entities (arthropods, traits and values) are recognised (identified in the texts) and normalised (mapped to dictionaries of known entities, if possible) and then the underlying relationships between arthropods and traits and between traits and values are extracted (annotated) where possible. Different entity recognition systems were, therefore, implemented to automatically recognise arthropods (taxa), traits and values and then the recognised entities were normalised according to a set of terminologies. To identify the relationships between the recognised entities, a relationship extraction model was applied where each relationship connects exactly two entities and the relationship type is determined by the types of connected entities.

*Named Entity Recognition (NER)*: The NER steps employed and tested several Bidirectional Encoder Representations from Transformers (BERT)-based models, namely BERT-large-uncased (Devlin et al. 2019), RoBERTa-large (Liu et al. 2019) and the domain-adapted BioBERT (Lee et al. 2020) model initialised on the BERT model and further pre-trained on PubMed abstracts and PMC complete articles. For NER of value entities, pre-trained models were used from SpaCy (Montani et al. 2023) and quantulum3 (Mündler 2024), which are libraries that are specialised in flexible matching of measurements and their entities.

*Named Entity Normalisation (Linking) (NEN)*: To perform NEN, the OntoGene BioMedical Entity Recogniser (OGER) was used, OGER offers a set of tools for text mining and information extraction (Basaldella et al. 2017, Furrer et al. 2022). The normalisation (or linkage) of entities is achieved by flexible matching of the recognised entities with the curated arthropod and trait dictionaries described above.

*Relationship Extraction (RE)*: The Transformer-based LUKE (Yamada et al. 2020) model was used for the RE task. The LUKE model takes a text string along with the offsets of a head and tail entity to perform classification according to a set of relationship labels.

### Technical Specifications of the ArTraDB Web Resource

ArTraDB is a web application designed and built to present the predicted annotations to the scientific community. The following technology stack was used to build the resource: data are stored in a Neo4J database (https://neo4j.com/) and made available through a backend Application Programming Interface (API) based on express.js (https://expressjs.com); the front-end was built using Vue.js (https://vuejs.org) and node.js (https://nodejs.org); for visualisation of annotated documents the TextAE annotation editor (https://textae.pubannotation.org) (Lever et al. 2020) was integrated into the web application.

## Results

### A Workflow for Annotating Arthropod Organismal Traits

**Figure 1.**
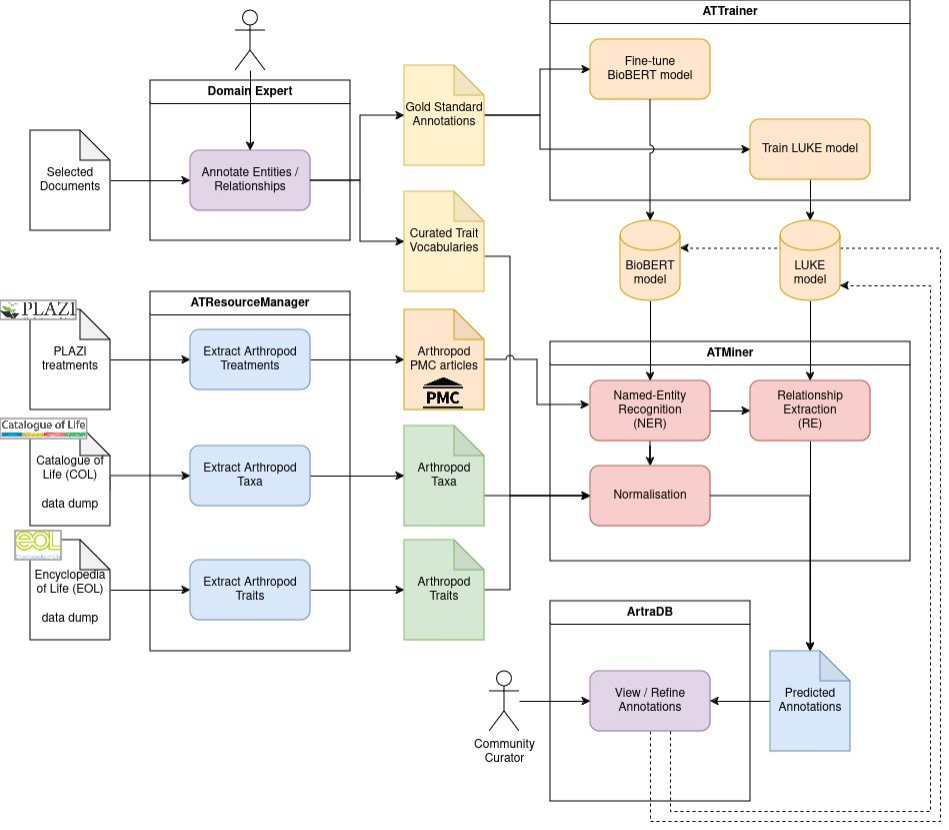
The arthropod organismal traits annotation workflow. The workflow starts with curation performed by domain experts resulting in entity and relationship annotations for a selected subset of publications as well as curated vocabularies of sets of organismal traits. The ATResourceManager steps include the processing of data sourced from the Catalogue of Life (taxonomy) and the Encyclopedia of Life (arthropod-trait relationships) to generate taxon and trait dictionaries, as well as the retrieval of publications for processing from PubMed Central, based on the selection of all Plazi TreatmentBank records for arthropods. The expert-generated Gold Standard Annotations are used as input to train (ATTrainer steps) Natural Language Processing (NLP) models for the Named Entity Recognition (NER) and Relationship Extraction (RE) tasks, with the trait vocabularies and taxa dictionaries being used for entity normalisation (ATMiner steps). Finally, the predicted annotations are made available to the scientific community via the ArTraDB web resource where community curators could potentially provide corrections to the annotations that can later be used for refinement of the NER and ER models (dotted lines).

The analytical workflow for processing and annotating thousands of articles to identify organismal traits of arthropods (Fig. 1) consists of several key data preparation steps (ATResourceManager and Domain Expert curation) and model training procedures (ATTrainer), in order to subsequently perform the text mining tasks (ATMiner) to produce the predictions and upload them for viewing in ArTraDB. Firstly, the domain expert curation tasks resulted in two key outputs: the Gold Standard Annotations and the Curated Trait Vocabularies. A set of selected documents was manually annotated by domain experts to provide a resource for downstream training and for assessing the performance of the text mining tasks (see Methods). The domain experts also built curated trait vocabularies (including synonyms) covering the three categories of feeding ecology (n = 81), habitat (n = 184) and morphology (n = 125), based on combinations of existing ontologies and online resources (see Methods). In parallel, the ATResourceManager preparation steps were developed to: (1) process the taxonomic treatment documents from Plazi and retrieve the corresponding publications from PMC; (2) extract from the Catalogue of Life taxonomy all accepted arthropod species and their higher-level taxonomic names; and (3) extract from the Encyclopedia of Life traits database all available taxon-trait annotations for arthropods (see Methods for details). Subsequently, the ATTrainer language model training steps take as input the Gold Standard Annotations (TRAIN-GOLD subset) for the fine-tuning of the BioBERT model and for the training of the LUKE model (see Methods). These models are then used in the ATMiner tasks for Named Entity Recognition (NER) with BioBERT and Relationship Extraction (RE) with LUKE, also using the curated trait vocabularies to perform entity normalisations using OGER (see Methods). The resulting predicted annotations - the entities of arthropods, traits and values - and the arthropod-trait and trait-value relationships were then imported into the ArTraDB web resource where they can be reviewed by the community.

### Entity and Relationship Annotation of PubMedCentral Articles

#### Annotation Results for Entity and Relationship Discovery

**Figure 2.**
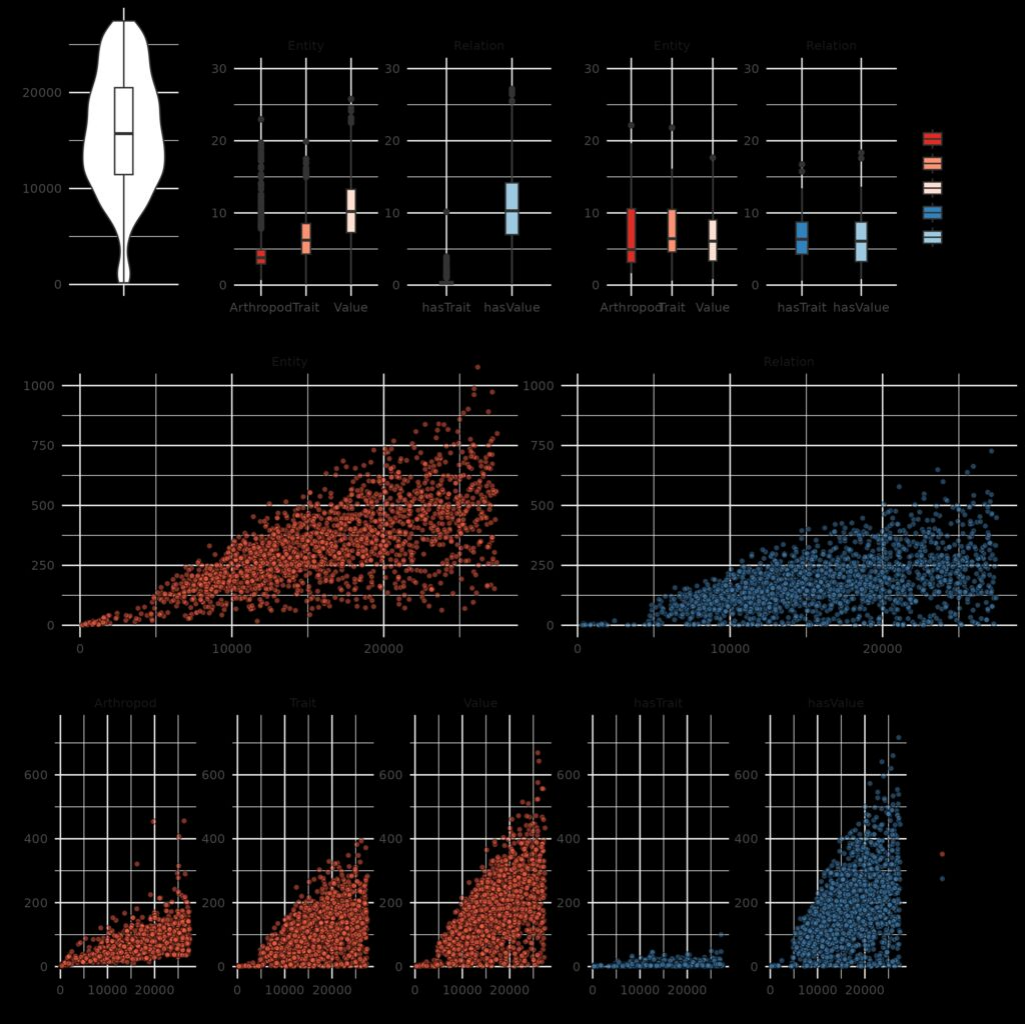
Distributions of PMC article properties and the resulting entity and relationship annotations. **(A)** The input dataset consisted of 2,000 PMC articles exhibiting a broad character (chars) length distribution. The relative number of resulting predictions of annotated entities (arthropods, traits, values) and relationships (taxon to trait - hasTrait, trait to value - hasValue) are shown for the whole dataset **(B)** and for the 25 gold-standard manually annotated documents **(C)**. The absolute number of predicted entity and relationship annotations compared to the document lengths in characters is shown for annotation types (D) and subtypes **(E)**. Boxplots in panels **A, B** and **C** show the median, first and third quartiles and lower and upper extremes of the distribution (1.5 × Interquartile range).

The application of the workflow presented in Fig. 1 to a total of 2,000 publications sourced from PMC resulted in the annotation of 656,403 entities (arthropods, traits and values) and 339,463 relationships (hasTrait, hasValue), summarised in Fig. 2. The PMC articles range in lengths from 173 to 27,466 characters with a median of 15,720 and an interquartile range from 11,452 to 20,506 (Fig. 2A). The densities of entity and relationship predictions are highest for entities of type “value” and hasValue relationships, with medians of ~ 10 annotations per 1,000 characters, arthropod and trait entities having median densities of four and six annotations per 1,000 characters respectively and hasTrait relationships have the lowest density with a median of zero annotations per 1,000 characters (Fig. 2B). In contrast, the 25 articles comprising the gold standard annotation dataset show median densities of 4.9, 6.4 and 6.1 annotations per 1,000 characters for arthropod, trait and value entities, respectively, and the hasTrait and hasValue relationships show medians of 6.4 and 6.1 annotations, respectively (Fig. 2C). These manually annotated documents (17 of which were complete articles and eight only abstracts) contained in total 4,990 named entities (1,069 arthropods, 2,078 traits and 1,843 values) and 3,628 relationships (1,777 hasTrait and 1,851 hasValue). For the predicted annotations, the total numbers of entities and relationships are generally higher in longer documents, reaching over 900 and over 600 annotations, respectively (Fig. 2D). This trend is replicated when considering each entity and relationship subtype separately, with the largest numbers of annotations identified in some of the longest documents, reaching maxima of 456, 393 and 669 for arthropods, traits and values and 100 and 717 for hasTrait and hasValue, respectively (Fig. 2E). At minimum, a publication should contain one taxonomic treatment describing a single arthropod species, for example, “ *Pachybrachissassii* a new species from the Mediterranean Giglio Island (Italy) (Coleoptera, Chrysomelidae, Cryptocephalinae)” (Montagna 2011) (length: 13,550 characters; annotated entities: 63 arthropod, 79 trait, 149 value). However, the much longer articles generally describe a whole group of species for a particular region, for example “The dipteran family Celyphidae in the New World, with discussion of and key to world genera (Insecta, Diptera)” (length: 27,381 characters; annotated entities: 172 arthropod, 141 trait, 246 value) contains 92 taxonomic treatments (Gaimari 2017).

#### Assessing the Complexity of the Task by Examining Inter-Annotator Agreement

**Figure 3.**
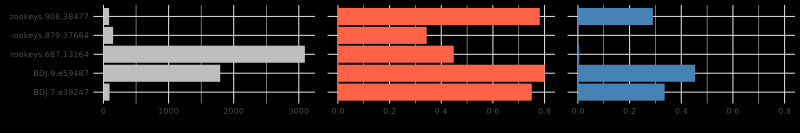
Inter-annotator agreement of five documents annotated by both experts. **(A)** The bars show document lengths in characters (chars); **(B)** The bars show the level of annotation agreement for entities (taxon, trait or value) between the two annotators as measured using Cohen’s kappa; **(C)** The bars show the level of annotation agreement for relations (hasTrait or hasValue) between the two annotators as measured using Cohen’s kappa.

To begin to interpret the prediction results from the workflow it is important to understand the complexity of the annotation task itself, insights into which can be gained by examining the levels of agreement between the curated annotations generated by the two domain experts. The five documents that were annotated by both domain experts included two complete publications and three abstract-only articles (Fig. 3). In total, for these five documents, the two experts annotated 1,477 named entities (161 arthropods, 764 traits, 552 values), with annotator 1 identifying 80 arthropods, 416 traits and 334 values and with annotator 2 identifying 81 arthropods, 348 traits and 218 values. They also annotated a total of 1,094 relationships (553 hasTait, 541 hasValue), with annotator 1 identifying 343 hasTrait and 343 hasValue relationships and with annotator 2 identifying 210 hasTrait and 198 hasValue relationships. Cohen’s kappa is used to measure inter-annotator reliability, or concordance, to assess the degree of agreement amongst independent observers who rate, label or classify the same phenomenon, with values below 0.6 generally indicating inadequate agreement (McHugh 2012). Amongst the five documents curated by both annotators, Cohen’s kappa values reflect varying levels of inter-annotator agreement for entities (Fig. 3B), from poor agreement (~ 0.35), to moderate agreement (~ 0.5), to substantial agreement (~ 0.8), with lower agreement levels for relationships (Fig. 3C). While Cohen’s kappa scores provide a standardised measure of agreement, their values must be carefully interpreted within the study’s context, where here they serve to highlight the complexity of the annotation tasks.

#### Assessing Entity Normalisation with the Taxon and Trait Dictionaries

**Figure 4.**
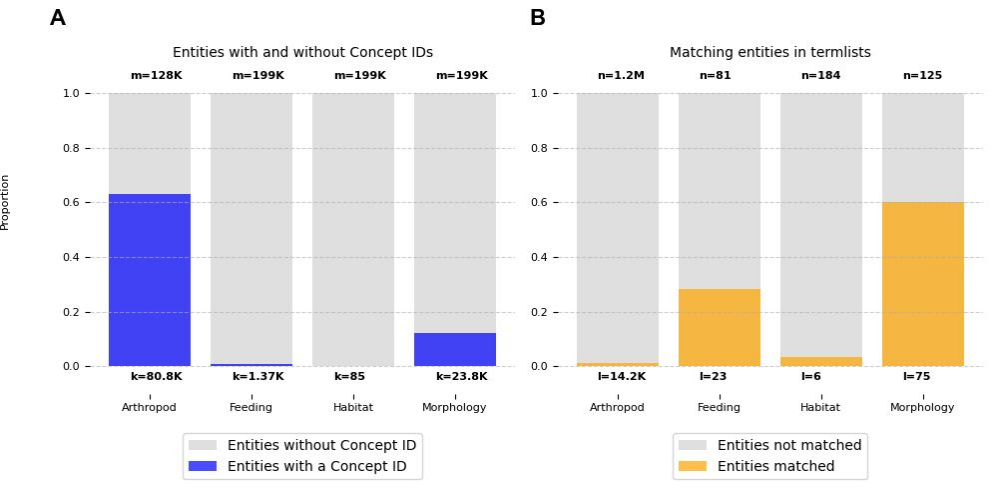
Taxon and trait dictionaries compared with annotated entities. For the 2,000 PMC articles analysed: the proportions of all annotated entities that could be mapped to the corresponding “Concept IDs” of the taxon and trait dictionaries (**A**) and the proportions of taxon and trait dictionary terms that were matched with annotated entities in any article (**B**). In **(A)**, ‘m’ represents the total numbers of taxon and trait entities and ‘k’ indicates how many of these were mapped to Concept IDs in the dictionary term-lists (for trait entities divided into feeding ecology, habitat and morphology). In **(B)**, ‘n’ represents the total numbers of taxa and traits in the dictionary term-lists and ‘l’ indicates how many of these were matched in the articles.

Entity normalisation, or linking, which was performed using OGER, is the process of matching the entities that were annotated in the articles with the dictionaries of arthropods (taxa from the Catalogue of Life) and traits (the collated sets of feeding ecology, habitat and morphology traits), with the goal of assigning to each labelled entity a unique identifier from one of the input resources. It is important to understand the performance of the normalisation task in order to interpret the quality of the entity prediction results from the workflow. The taxon dictionary contained a total of 1,015,642 arthropod species and 118,008 higher-level taxonomic names and the trait dictionary contained a total of 390 traits: 81 feeding ecology; 184 habitat; 125 morphology (see Methods). Focusing on the identification and quantification of taxon and trait entities within the article corpus, the coverage and frequency of entities mapped to the predefined dictionaries and those that could not be mapped provide an assessment of the entity normalisation process and the comprehensiveness and relevance of the dictionaries (Fig. 4).

Across the 2,000 articles processed by the workflow, a total of 128,149 taxon entities were annotated with 63% matching terms in the dictionary (mapped to Concept IDs), comprising 24,207 species and 56,532 higher-level taxonomic names (Fig. 4A). Notably, taxa, such as the order Hymenoptera (sawflies, wasps, bees and ants) or the genus Tipula (crane flies), were amongst the most frequently annotated entities, with 388 and 384 occurrences, respectively. Reviewing some of the 47,312 non-mapped taxon entities revealed examples of correctly identified arthropod taxa which, nevertheless, are not included in the Catalogue of Life and are, therefore, not in the dictionary, for example, the genera Micrencaustes (beetles) and Deinodryinus (parasitoid wasps). Of the 199,276 (28,348 unique) trait entities annotated, 12.7% were successfully mapped to the trait dictionary (linked to Concept IDs), with 1,366, 85 and 23,816 entities mapping to feeding ecology, habitat and morphology terms, respectively (Fig. 4A). Note that, as the NER task labels entities as taxa, traits or values, further categorisation of traits into feeding ecology, habitat and morphology terms is only possible when entity normalisation is successful. Feeding and morphology traits such as “host” (273), “mouth” (16) and “legs” (1,663) were amongst the most prevalent annotated entities. Many annotated trait entities could not be mapped (168,237), one of the most frequent being “distribution”, with 2,506 occurrences. Considering instead the numbers of unique terms in the dictionaries that could be annotated and linked in the articles, only 14,243 of the 1.2 million arthropods in the dictionary (1.2%) were identified and, from the trait dictionary of feeding ecology, habitat and morphology terms, 28.4%, 3.3% and 60%, respectively, were annotated and linked (Fig. 4B). Failure to identify dictionary terms in the annotated articles may be because the terms are simply not present (it cannot be expected that a small subset of 2,000 articles will contain mentions of all 1.2 million described arthropod species) or because normalisation was unable to link terms and phrases recognised as entities in the texts with the terms, phrases and synonyms that make up the concepts of the dictionaries.

### Performance Comparisons of Natural Language Processing Models

#### Named Entity Recognition Baseline Performance

**Figure 5.**
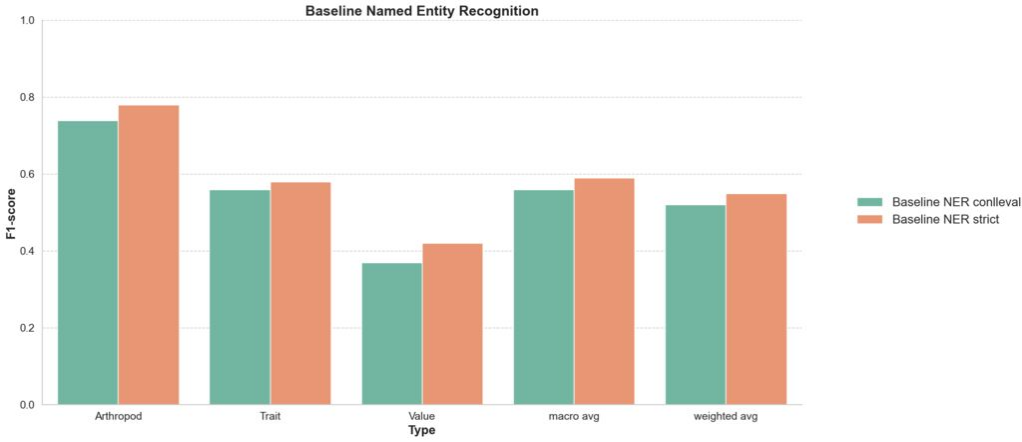
CoNLL evaluation and strict F1-score baseline results for the named entity recognition. The F1-score performance of the baseline Named Entity Recognition (NER) model on the test set of PubMedCentral (PMC) articles using the Conference on Natural Language Learning (CoNLL) evaluation (conlleval) and strict methods. The scores are shown explicitly for three entity types: Arthropod, Trait, and Value. Additionally, for the three types combined, the macro-average F1-score, which does not account for class imbalances and the weighted average F1-score, which adjusts for the imbalance of different classes, are also presented. A detailed listing of all baseline results can be found in Suppl. material 4.

An evaluation of a Named Entity Recognition (NER) baseline was conducted across various configurations. Several general and domain-specific pre-trained language models were fine-tuned on the TRAIN-GOLD dataset. To train the models, the dataset was converted to IOB2 format. Two evaluation methods were employed for the results presented in Fig. 5: the Conference on Natural Language Learning (CoNLL) evaluation and strict metrics. The reported results are based on the F1-score (F) and corresponding Precision (P) and Recall (R). Under the CoNLL evaluation, the baseline demonstrated a macro-average 0.56 F (0.55 P / 0.57 R) and a weighted-average of 0.52 F (0.53 P / 0.53 R) across all entity types. Notably, entities classified as ‘Arthropod’ achieved the highest F1-score at 0.74 F (0.7 P / 0.78 R), signifying superior recognition capabilities in comparison to other categories. Conversely, ‘Value’ entities posed greater challenges, with the lowest score of 0.37 F(0.32 P / 0.43 R). This indicates substantial difficulties in the precise identification of these entities. ‘Value’ entities encompassed a diverse array of concepts, ranging from measurements (e.g. ‘56.6 mm’) and colour descriptors (e.g. ‘brownish-yellow’) to locations (e.g. ‘China’). This disparity highlights the model’s varied performance across different entity types. When evaluated using the strict metric, a notable enhancement in both precision and F1-scores was observed for most entity types, compared to the CoNLL evaluation metric (Fig. 5). ‘Arthropod’ entities maintained the highest score 0.78 F (0.78 P / 0.78 R), consistent with the previous evaluation. The overall macro- and weighted-average scores increased to 0.59 F (0.63 P / 0.57 R) and 0.56 F (0.6 P / 0.52 R), respectively, indicating a more accurate entity recognition when the strict metric was applied. This comparison not only underscores the baseline’s strengths and weaknesses in recognising various entities, but also highlights the impact of evaluation criteria on perceived performance.

#### Relationship Extraction Baseline Performance

**Figure 6.**
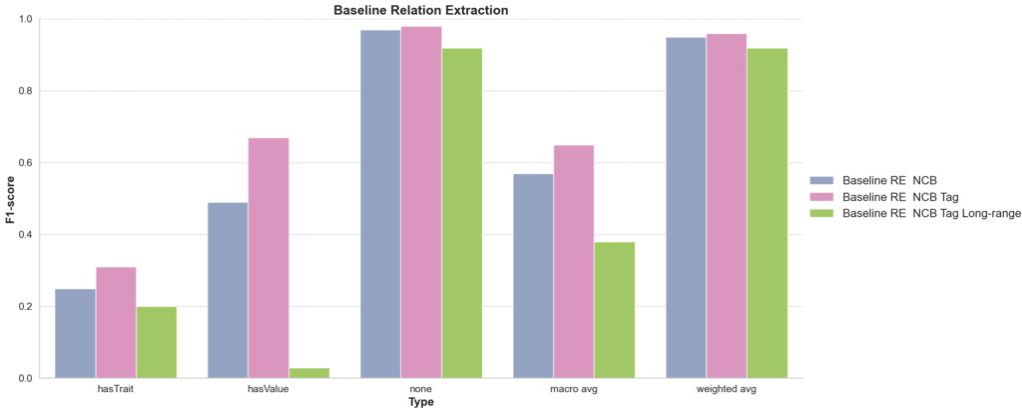
NONE-class balanced (NCB), entity tagged (Tag) and long range baseline results for relationship extraction. The F1-score performance of three baseline configurations of the Relationship Extraction (RE) model on a test set of PubMedCentral (PMC) articles. The configurations are: Non-Class-Balanced (NCB), which limits the amount of ‘none’ relationships during training to match the majority class; XML Inline Tag Entities (Tag); and Long-range Context, which provides a context of 250 characters around each target entity rather than using a sliding context window. Performance scores are specifically shown for two relationship types: ‘hasTrait’ between Arthropod and Trait, ‘hasValue’ between Trait and Value and ‘none,’ which indicates the absence of a relationship. Additionally, the figure includes both the macro average F1-score, which does not account for class imbalances and the weighted average F1-score, which compensates for these imbalances. A detailed listing of all baseline results can be found in Suppl. material 4.

Fig. 6 outlines the outcomes of the Relationship Extraction (RE) baseline across three different configurations of the LUKE model, namely “NCB” (None-Class Balanced), which limits the amount of ‘none’ relationships during training to match the majority class, “Tag” which uses XML to tag the entities inline and “Long-Range”, which captures long-range relationships via a different training set-up. By default, the LUKE model was used with a shifting context window spanning 1 - 6 consecutive sentences to detect relationships. In contrast, for the Long-range approach a version of the LUKE model was fine-tuned by extracting and merging the two target entities with their 500 surrounding characters each. The NCB approach, even after balancing the frequency of ‘none’ classes with the most common relationship (‘hasValue’), continued to face challenges in accurately identifying specific relationships like ‘hasTrait’. This indicates persistent difficulties in detecting nuanced or less common relationships. Furthermore, applying the Tag approach in addition to the NCB approach improved the RE baseline in the macro-average score from 0.57 F (0.62 P / 0.66 R) to 0.65 F (0.66 P / 0.69 R) compared to the standard NCB configuration. This suggests that entity tag information contributes positively to relationship extraction performance. Using NCB and Tag combined with the Long-range RE set-up demonstrated an interesting pattern with ‘hasValue’ relationships, where a perfect recall (1.00), but very low precision (0.02) resulted in a low F1-score (0.03). This indicates the model’s tendency to over-identify ‘hasValue’ instances, leading to numerous false positives. The prediction results for the ‘hasTrait’ relationship show 0.2 F (0.13 P / 0.52 R), while scoring for ‘none’ relationship 0.92 F (1.0 P / 0.86 R) and a macro-average of 0.38 F (0.38 P / 0.79 R), making it the worst-performing baseline.

### The Arthropod Trait Database ArTraDB Web Resource

The annotation predictions obtained from applying the workflow to the PMC articles are made available to the community through the dedicated web application, ArTraDB: the Arthropod Trait Database (https://artradb.unil.ch). The results are presented in a simple table-like view where each row represents a single entity annotation, pairs of entities connected by either a hasTrait or hasValue relationship or complete trio annotations of connected Arthropod-Trait-Value entities. The ArTraDB resource was designed and developed to provide two main functionalities: (i) Browse and search facilities enabling the identification of predicted species/taxa and/or traits and/or values within the set of annotated documents; and (ii) Browsable visual displays of the predicted entity and relationship annotations in the local context of the corresponding document.

#### ArTraDB Browse and Search Functionalities

**Figure 7.**
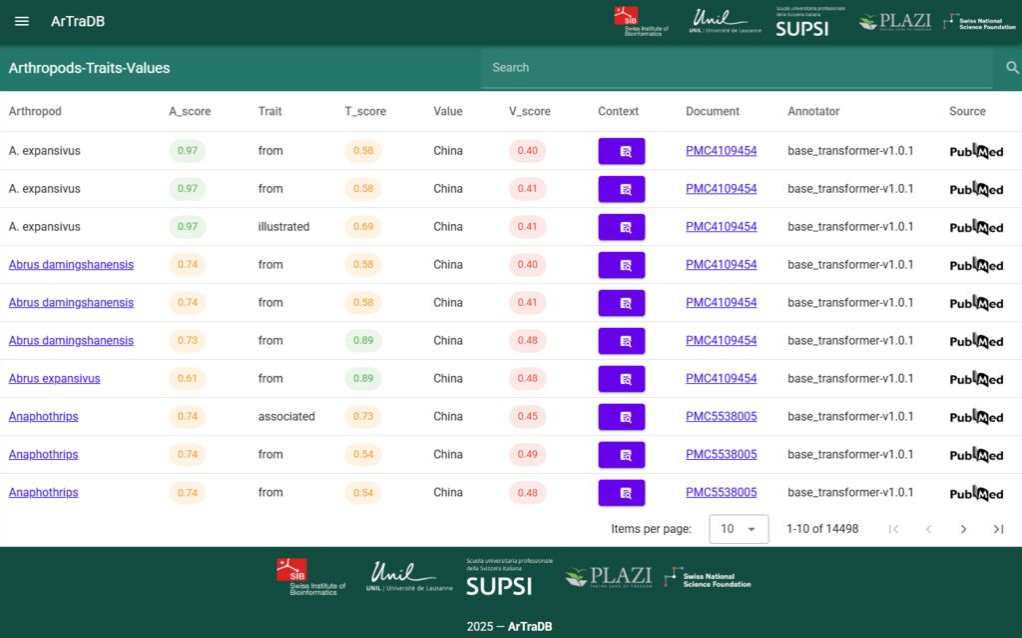
ArTraDB table view browse and search functionalities. The table view of annotations displays rows of annotated entities with their corresponding Named Entity Recognition (NER) confidence scores, a clickable icon to open the Annotation Viewer window showing the local context of the annotations, the hyperlinked PubMedCentral (PMC) article identifier to view the fully annotated article (Annotator) and the PubMed icon to link to the source publication, in a browsable paginated format with a user-configurable number of items to display per page. Annotated arthropods and traits that were successfully linked to Concept IDs in the corresponding dictionaries are hyperlinked to the corresponding source definition, for example, the Catalogue of Life (COL) for arthropods and the Encyclopedia of Life (EOL) or other resources for trait entities. Indexing of the ArTraDB data allows for rapid user searches to filter the complete table to rows with entries matching terms entered in the simple search box above the table.

The browsable table view of the workflow-predicted annotations for the set of processed articles provides a paginated display of rows of annotated entities together with the NER confidence scores assigned by the prediction algorithm (Fig. 7). Where both hasTrait and hasValue relationships connect an arthropod entity with a trait entity and that same trait entity to a value entity, the row represents a complete Arthropod-Trait-Value trio annotation. When either hasTrait or hasValue relationships are lacking, the row displays only the Arthropod-Trait or Trait-Value pairs. If no relationships were predicted, the arthropod and trait entities are displayed as single annotations with their corresponding scores, while the value entities are omitted. When entity normalisation was able to successfully link annotated arthropods and traits to Concept IDs in the corresponding dictionaries, these are hyperlinked to the corresponding source definition, for example, the Catalogue of Life for arthropod entities and the Encyclopedia of Life or ontology and Wiki resources for trait entities. Additional columns in the table view include a clickable icon to open the popup Annotation Viewer window, the PMC identifier hyperlinked to the fully annotated document, the source of the annotation (i.e. version of the workflow that made the predictions). The table view can be browsed page by page with a user-configurable number of rows to display per page. The data in each column are indexed to enable rapid user searches using the simple search box above the table that filters the results to contain only rows matching the entered search term. This simple table view of the annotations provides an intuitive browsable and searchable interface to the thousands of annotations produced by the workflow.

#### ArTraDB Document Context View of Annotations

**Figure 8.**
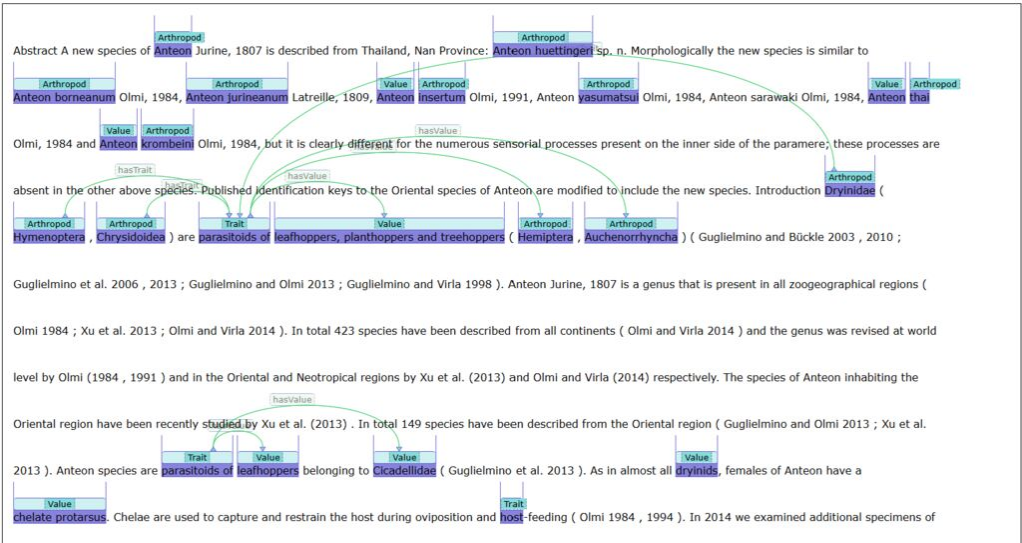
ArTraDB document view for visualising all predicted annotations in an article. Clicking the hyperlinked PubMedCentral (PMC) identifier in the ArTraDB table view opens a document view of the fully annotated article showing all entities and relationships. The entities are highlighted and labelled as Arthropod, Trait or Value entities, with the predicted relationships indicated by lines connecting the relevant entities. This provides users with the full-text context of each predicted entity or relationship annotation within the document.

For each row of predicted entity or entity and relationship annotations, a clickable icon in the Context column allows users to open an annotation viewer window that shows these labelled entities and any predicted relationships in the local context of the source text. Alternatively, clicking the hyperlinked PMC identifier opens a document view of the fully annotated article showing all entities and relationships. In both viewers, the entities are highlighted and labelled as Arthropod, Trait or Value entities, with the predicted relationships indicated by lines connecting the relevant entities (Fig. 8). These visualisation functionalities allow users to view the predictions in their local and global contexts, to be able to manually assess the reliability of the automatically generated annotations.

## Discussion

Focusing on the methodological explorations of the opportunities presented by modern literature mining approaches, we aimed to annotate available arthropod organismal trait data to facilitate the automated extraction of knowledge from publications. This involved building and testing an analytical workflow for processing and annotating thousands of PMC articles containing taxonomic treatments of arthropods. It also required the collation of curated trait vocabularies and the development of model training procedures to perform the text mining tasks and formally test the performance of various approaches. Finally, the resulting predicted annotations of the entities of arthropods, traits and values, as well as the arthropod-trait and trait-value relationships were made available for community review through the deployment of the open online ArTraDB web resource. Here, we discuss the key findings and important challenges encountered while exploring the utility and performance of the different methodologies and approaches we tested to achieve our aims.

### Results are variable because annotation is an inherently complex task

The process used to annotate the articles and train the models presents several methodological challenges that influence the efficiency and accuracy of predicting arthropod-trait-value triples. A key challenge was the generation of training data through the manual annotation of documents by domain experts. Compared to the predictions, the annotators identified higher median densities of arthropod and trait entities and hasTrait relationships, while the automated processes produced higher median densities for value and hasValue annotations (Fig. 2). Nevertheless, assessing inter-annotator agreements highlighted the complexity of the annotation tasks and the challenge of uniformity in defining entity boundaries or relationships between entities (Fig. 3). Defining entity boundaries and relationships in complex cases are subjective decisions that can diverge, based on the annotator’s understanding and experience. For example, “*mesoscutum silvery setae evenly distributed*”: could be simply annotated as trait=“*mesoscutum*” and value=“*silvery setae evenly distributed*” or by introducing a discontinuous trait “*mesoscutum setae*” with two associated values “*silvery*” and “*evenly distributed*”. The variability in manual annotations introduces conflicts in the input data that serve as the basis for training the NER and RE models, which, therefore, negatively impacts the model performance. While independence is required to formally assess inter-annotator agreement for such tasks, the recommendation from this work is to build a set of guidelines using examples encountered in the documents. The guidelines represent the consensus of the domain experts on how to proceed technically with annotating complex cases and, therefore, serve to enhance consistency and ultimately provide better input data for training the models. Indeed, guidelines and annotator training in the context of constructing NER and RE benchmarking corpora of abstracts and metadata files from biodiversity datasets achieved inter-annotator entity agreements of 0.76 and 0.70 for two pairs of annotators (Abdelmageed et al. 2021). As well as the manual annotation variability, the low amount of training data - the 25 articles comprising the gold standard annotation dataset - also posed challenges. One strategy to increase the amount of training data could be to use distant supervision techniques, such as employing large language models (LLMs) to generate structured annotated texts, based on the manually-annotated examples (Li et al. 2024). This could introduce biases and would likely be limited to shorter text bodies and smaller annotation context ranges than those encountered in the published articles, but the approach remains worth considering to augment training data. Ultimately, more consistent and better quality annotations across many more articles could be achieved through community review of the NER and RE predictions. The integration of such community-reviewed annotations into future training datasets (Fig. 1) could, therefore, serve to iteratively improve model performance with a growing corpus of articles in the gold standard annotation dataset.

### Entity recognition performs better than relationship extraction

The NER and RE tasks present distinct challenges as evidenced by the performance disparities in the baselines (Figs 5, 6). NER focuses on identifying and classifying entities in the text, with results showing a varied performance across different entity types, where “Arthropod” entities were consistently easier to correctly recognise than “Value” entities and “Trait” entities showing intermediate performance (Fig. 5). In contrast, RE aims to identify relationships between entities, which is an inherently more complex task due to the need to understand context and entity interactions where the entities themselves may not be correctly or completely identified. The results indicate that, while the baseline is proficient in identifying the absence of a relationship (“none”), it struggles with more specific and low-frequency relationships. The balancing of classes in the training and the introduction of entity tags slightly improved the performance, but also revealed the model’s limitations in generalising across different types of relationships (Fig. 6). Of particular interest with respect to the processing of taxonomic treatments is the question of context sizes when attempting to identify entity relationships. The hasTrait relationships can often be long-range and few-to-many because the arthropod may be mentioned a few times near the start of an article where the text that follows is then dense with many specific trait entities, for example, a paragraph with detailed descriptions of the morphology of the arthropod. In contrast, the hasValue relationships are more often likely to be short-range and one-to-one as the values of the mentioned traits are usually presented in very close proximity (same sentence). The close proximity can still present substantial challenges, for example, in taxonomic treatments where lists of traits are followed by corresponding lists of values: “*Antennal segments III–VIII length 38, 47, 43, 41, 33, 21*” (here with the added complexity of an inferred list of segments III, IV, V, VI, VII and VIII). Given these differences, alternative strategies where training and RE steps are carried out separately for hasTrait and hasValue relationships might perform better. Overall, the distinction between the NER and RE tasks is evident here in their respective challenges and baseline performances. NER requires accurate classification of individual entities, while RE demands a deeper understanding of the context and the interactions between entities. This difference in complexity was also reflected in the lower inter-annotator agreement scores achieved for RE than for NER annotations (Fig. 3), which would have reduced the effectiveness of training the LUKE model. While the use of NER and RE in mining biodiversity literature has shown promising results, the complexities associated with training data quality and annotation consistency remain substantial challenges. Addressing these issues will be essential for advancing the capabilities of NLP applications in biodiversity research.

### Entity normalisation is a critical yet challenging process

The task of normalisation involves linking the identified entities to defined dictionaries, vocabularies or ontologies with accompanying descriptions and associated information. Without linking, the annotation simply represents a hypothesis that a given entity can be classed as either an arthropod, a trait or a value. Taxonomic names are structured terms deriving from academic consensus and they are also relatively consistent and used in a similar format in most publications; therefore, normalisation should usually be feasible. Indeed, 63% of taxon entities matched terms in the dictionary (Fig. 4), i.e. they could be linked to species and higher-level taxonomic names from the Catalogue of Life (COL). This result is promising when compared to the performance of other NER systems developed specifically for taxonomic name recognition, which, depending on the corpus used for assessment, can range in precision from 23% to 96% (Le Guillarme and Thuiller 2022). In preparing the arthropod taxa dictionary, only COL accepted names were considered (see Methods); however, given the diversity of sources and ages of the processed publications, a universal taxon dictionary including all ever-recorded names and synonyms would have resulted in higher normalisation levels. A more comprehensive taxon dictionary could instead be sourced from COL and GBIF’s ChecklistBank repository for taxonomic datasets (Döring et al. 2022). With respect to traits, normalisation levels were considerably lower than for taxa, reflecting the much less structured manner in which traits are usually described in publications, as well as the variability of terms used across different groups of species (Fig. 4). This can be further complicated by the use of a single term to refer to different concepts (usually morphological) or, indeed, the use of different terms to refer to the same (homologous) structures, in different taxanomic groups. Of the three categories of traits, morphology achieved the highest level of linking, likely explained by the expectation for taxonomic treatment publications to be dense in morphological descriptions that are used to define and distinguish between species. While nearly a third of the feeding ecology traits from the dictionary could be linked to trait entities in the documents, this represents only a small fraction of all annotated trait entities. In contrast, linking of habitat traits proved very challenging, with only six terms being linked to annotated trait entities. This likely reflects considerable differences between the formal style of term names used by the Environment Ontology and the more variable descriptions of habitats used in natural language. In summary, while entity normalisation remains challenging, iterative extension, refinement and curation of the dictionaries should lead to higher levels of linking, for example, by expanding the taxon dictionary to include all known names and synonyms and by extending, revising and curating the feeding ecology vocabulary and habitat ontology to add synonyms and additional terms that align better with natural language usage. Additionally, while this work focused on arthropod organismal traits, enhancing the trait dictionaries – which are key for the normalisation process – by aligning them with other existing ontologies developed for biodiversity research more generally, such as the BiodivOnto (Abdelmageed et al. 2021), could improve both entity recognition and the linking of entities to formalised concepts.

### Integrating community feedback into future ArTraDB functionalities

In addition to enhancing the breadth and utility of the trait dictionaries and the performance of the workflow, future work will also need to extend the set of gold standard annotations to improve NER and RE performance through better training and benchmarking. An important source of new annotations could be achieved through further developing the ArTraDB resource functionalities to allow user curation of the workflow-predicted annotations. This would require integration of curation tools into the Annotation Viewer window (Fig. 8) that allow community users to edit, add, confirm or delete entity and relationship annotations. This would facilitate “community curators”, i.e. scientifically literate individuals, but not necessarily domain experts, to confirm or modify the annotations, as well as deleting wrong or adding missing annotations, in a crowd-sourcing-like operation. The development of this functionality opens up opportunities to then incrementally fine-tune the prediction models by providing them with human-validated annotations as additional training data. While technically feasible and indeed prototyped in the development version of ArTraDB, before deploying such an interactive functionality, it would be important to first build the infrastructure for the long-term preservation of community-sourced curated annotations. This could involve the publication of community-confirmed entities and relationships to infrastructures such as the Encyclopedia of Life or Wikidata; however, this is technically challenging and would only cover normalised entities. Alternatives could be to publish Journal Article Tag Suite (JATS) XML and/or BioC JSON files on repositories like Zenodo (CERN and OpenAIRE 2024) or even to archive individual annotations as nanopublications with detailed provenance information to identify the source document and location within the document. A more comprehensive approach might instead be to focus on infrastructures supporting the central indexing of biodiversity-related literature (Pasche et al. 2023a, Pasche et al. 2023b) that facilitate the addition of annotations to the articles using JATS XML and/or BioC JSON formats. Once a sustainable solution is in place, then ArTraDB could begin to collect community feedback for use as part of improved training and benchmarking datasets and for collation into versioned annotation sets for archiving or integration into open biodiversity literature services.

### Perspectives on literature mining for biology, ecology and evolution research

Our methodological explorations to develop tools and resources to advance the use of text mining approaches in biology, ecology and evolution research, demonstrate the feasibility of semi-automating the building of open databases of organismal traits extracted from the literature. Even if the annotated arthropod taxon-trait-value triples are sparse, they enable researchers to quickly locate documents pertaining to specific species and traits. This not only accelerates the initial stages of data curation, but also points researchers to the exact locations within documents where relevant data can be found, thereby having the potential to enhance the efficiency of research workflows. These advances have the potential to greatly enhance taxonomic resolution and representation in trait-based studies and trait databases, such as those compiled for groups including ants, bees, beetles, butterflies, spiders and others (Parr et al. 2016, Wong et al. 2019, Pekár et al. 2021). Given the specificities within different arthropod groups, taxonomically-tailored extensions to the trait dictionaries could further enhance the reliability and utility of semi-automated trait annotations for augmenting such databases. While there remain several technical challenges to overcome, including how best to leverage the power of modern LLMs in these processes (Farrell et al. 2024, Marcos et al. 2024, Keck et al. 2025), the results provide a framework that could be extended beyond the focus on arthropods. These and other TDM and NLP initiatives in the biodiversity domain will enhance data synthesis studies, make literature reviews more reproducible, greatly facilitate identification of research knowledge gaps and biases, as well as drive data-informed investigations of ecological and evolutionary trends and patterns (Farrell et al. 2022, Farrell et al. 2024). Improving the taxonomic foundation and diversifying the accessible data should also contribute to advancing the applications of trait-based approaches to understanding community ecology, (multi-)trophic interactions and predicting patterns in biodiversity and ecosystem functions across space and time (Wong et al. 2019, Westoby 2024). When a trait or set of traits has been carefully curated and the relevant group of species is well-represented with genomics data, researchers can begin to ask how genetic and genomic changes relate to observable phenotypic differences, for example, swallowtail butterfly lineages with host-plant shifts have more genes under positive selection than non-shifting lineages (Allio et al. 2021), transitions to parthenogenesis (asexual reproduction) in stick insects are accompanied by greatly reduced genetic diversity and reduced rates of positive selection (Jaron et al. 2022) and dietary shifts of ladybird beetles from fungivory to carnivory and herbivory are accompanied by changes in chemosensory, digestive and immunity gene families (Huang et al. 2025). As genomics and other “omics” data become more accessible and as catalogues of species traits become more comprehensive – relying on automation for scale and curation for quality control – new opportunities for studying complex evolutionary processes will emerge (Cornwallis and Griffin 2024). There are also implications beyond fundamental research, for example, in the context of detecting and reporting biodiversity change globally, data and knowledge are critical for the measurement framework of Essential Biodiversity Variables (EBVs), where TDM and NLP tools and services could contribute especially to providing information for “species traits” EBVs (Kissling et al. 2018). While much of the knowledge about biodiversity collected and published over centuries remains largely not machine-readable, digitisation efforts and open-science initiatives are contributing to the opening up of biodiversity literature (Agosti et al. 2024) and natural history collections (Groom et al. 2023). Therefore, the continued development and enhancement of specialist and generalist biodiversity literature mining tools and resources is required to serve researcher needs, as well as to provide information for assessments and guide policy decisions on the protection and restoration of biological diversity for a sustainable future. Furthermore, efforts to enhance the annotation of legacy literature will need to be complemented by new solutions that enable publishing of biodiversity-related research in machine-actionable formats, facilitating seamless liberation of the data within.

## Supplementary Material

003C7273-A2CF-5CE9-9D0E-B2D3E68A84F8 10.3897/BDJ.13.e153070.suppl1 Supplementary material 1 Supplementary File S1 Data type traits Brief description An MS Excel spreadsheet presenting the lists of trait dictionaries for feeding ecology, habitat and morphology, with links to the source resources, synonyms and definitions. File: oo_1255917.xlsx https://binary.pensoft.net/file/1255917 Joseph Cornelius, Robert M. Waterhouse

0E178CB7-A073-5C16-B3CE-4C4B15FE3EBC 10.3897/BDJ.13.e153070.suppl2 Supplementary material 2 Supplementary File S2 Data type text Brief description A PDF file of the notes and guidelines developed by the annotators during the curation of the gold-standard annotation data. File: oo_1255919.xlsx https://binary.pensoft.net/file/1255919 Joseph Cornelius, Harald Detering, Robert M. Waterhouse

EA965717-4CF8-5A2B-9C61-9A50510914E5 10.3897/BDJ.13.e153070.suppl3 Supplementary material 3 Supplementary File S3 Data type text Brief description An MS Excel spreadsheet listing the annotated files, the number of annotations for each type and the corresponding annotators. File: oo_1255920.xlsx https://binary.pensoft.net/file/1255920 Joseph Cornelius

1E664958-F0A7-58CE-96D6-2691CF930F7C 10.3897/BDJ.13.e153070.suppl4 Supplementary material 4 Supplementary File S4 Data type performance metrics Brief description An MS Excel spreadsheet containing five tables with exact scores for all configurations, in terms of recall, precision and F-score values, along with the corresponding support for each class and the macro- and weighted averages. File: oo_1255921.xlsx https://binary.pensoft.net/file/1255921 Joseph Cornelius
